# The PIN family of proteins in potato and their putative role in tuberization

**DOI:** 10.3389/fpls.2013.00524

**Published:** 2013-12-19

**Authors:** Efstathios Roumeliotis, Bjorn Kloosterman, Marian Oortwijn, Richard G. F. Visser, Christian W. B. Bachem

**Affiliations:** ^1^Laboratory of Plant Breeding, Department of Plant Sciences, Wageningen UniversityWageningen, Netherlands; ^2^Group of Biotechnology of Pharmaceutical Plants, Laboratory of Pharmacognosy, Department of Pharmaceutical Sciences, Aristotersity of ThessalonikiThessaloniki, Greece; ^3^Keygene N.V.Wageningen, Netherlands; ^4^Centre for Biosystems GenomicsWageningen, Netherlands

**Keywords:** auxin, auxin transport, PIN genes, potato tuberization, organ development

## Abstract

The PIN family of trans-membrane proteins mediates auxin efflux throughout the plant and during various phases of plant development. In *Arabidopsis thaliana*, the PIN family comprised of 8 members, divided into “short” and “long” PINs according to the length of the hydrophilic domain of the protein. Based on sequence homology using the recently published potato genome sequence (*Solanum tuberosum* group Phureja) we identified ten annotated potato *StPIN* genes. Mining the publicly available gene expression data, we constructed a catalog tissue specificity of *StPIN* gene expression, focusing on the process of tuberization. A total of four *StPIN* genes exhibited increased expression 4 days after tuber induction, prior to the onset of stolon swelling. For two PIN genes, *StPIN4* and *StPIN2*, promoter sequences were cloned and fused to the GUS reporter protein to study tissue specificity in more detail. *StPIN4* promoter driven GUS staining was detected in the flower stigma, in the flower style, below the ovary and petals, in the root tips, in the vascular tissue of the stolons and in the tuber parenchyma cells. *StPIN2* promoter driven GUS staining was detected in flower buds, in the vascular tissue of the swelling stolons and in the storage parenchyma of the growing tubers. Based on our results, we postulate a role for the *StPINs* in redistributing auxin in the swelling stolon during early events in tuber development.

## Introduction

Auxin is involved in various developmental processes, such as flower development, root development, and embryo patterning (Rahman et al., 2007; Krizek, 2011; Mashiguchi et al., 2011). The presence of auxin in the plant cell and its differential effect on the cell in relation to cell division, growth and differentiation, is controlled by two determinants: the rate of turnover and transport. The main sites of auxin biosynthesis are the shoot apical meristems along with cotyledons, expanding leaves and roots (Ljung et al., 2001). From the sites of biosynthesis, auxin is transported to other parts of the plant by diffusion or through active transport. The directional Polar Auxin Transport (PAT) system distributes auxin from the sites of biosynthesis to lower parts of the plant and is mediated by influx and efflux carriers. The influx of auxin in the plant cells is mediated by the influx carriers *auxin resistant 1/like aux1* (AUX/LAX), while efflux carriers have been identified as the PIN family of proteins (Gälweiler et al., 1998; Marchant et al., 1999, 2002). The PIN family has been investigated extensively in *Arabidopsis thaliana*, rice, and tomato (Krecek et al., 2009; Miyashita et al., 2010; Pattison and Catalá, 2011). The asymmetric distribution of the influx and efflux proteins on the plasma membrane results in the directional movement of IAA from the upper to the lower parts of the plant.

In *A. thaliana*, the family *PIN* family comprised of 8 members (*AtPIN1–8*), and is divided into two groups. *AtPIN1* to *AtPIN4, AtPIN6* and *AtPIN7* represent the canonical (long) PINs. The long PIN proteins have a relatively long central hydrophilic loop and share high sequence similarity, especially in the hydrophobic domains of both N- and C- termini. *AtPIN5* and *AtPIN8* form the second group of the *PIN* family (“short” *PIN* genes). Short *PINs* lack the central hydrophilic loop. [PIN structure and evolution reviewed in Krecek et al. (2009)]. The first PIN mutant, *AtPIN1*, exhibited a naked, pin-forming inflorescence with no or just a few defective flowers indicating the importance of proper auxin localization and the role of PIN proteins in plant development (Gälweiler et al., 1998). *AtPIN2* and *AtPIN4* are involved in the gravitropic response and root development, respectively, (Muller et al., 1998; Friml et al., 2002) while *AtPIN3* has a role in gravitropic and phototropic responses as well as apical shoot formation (Okada et al., 1991). In contrast to the long PINs, which are located on the plasma membrane, the short AtPIN5 is localized on the endoplasmic reticulum and participates in the compartmental localization and homeostasis of auxin (Mravec et al., 2009). The intron/exon structure bears similarities within the *PIN* family members across plant species (Krecek et al., 2009). In *A. thaliana*, the typical example is consisted of six exons, and exceptions are found only in the short *PINs* or in *PINs* similar to *AtPIN2*.

In the *Solanaceae* family, the PIN family of proteins has only been studied in tomato where the role of the PIN proteins in auxin distribution during fruit development was evaluated (Pattison and Catalá, 2011). The SlPIN group comprised of ten members. Six of those are long PINs and SlPIN5, 6, 8, and 10 are short PINs. In potato, the *PIN* genes have not been functionally studied or systematically compared on a genome-wide scale. Two potato genes, highly homologs to *A. thaliana PIN* genes, were shown to be up-regulated in the stolon tips just prior to tuber swelling and down regulated soon after (Kloosterman et al., 2008). First evidence of a role for auxin in tuber development was provided by the finding that an *Auxin Responsive Factor* (*ARF6*) was shown to exhibit a differential expression profile during early stages of tuber development (Faivre-Rampant et al., 2004). In addition, investigation of the auxin content during several early stages of tuber development revealed that auxin content increases locally in the stolon tip just prior to stolon swelling (Roumeliotis et al., 2012). These results indicate prominent roles for auxin and auxin transport in the regulation of potato tuberization, although a coherent sequence of events still has to be established.

In this paper, we describe the identification of additional *PIN* gene family members using the recently published potato genome sequence. Furthermore, we used available RNA-seq data to study variation of *StPINs* expression in a number of potato tissues. In addition, we studied the expression of all the *StPINs* during the early stages of tuberization using quantitative RT-PCR. Finally, we studied the expression pattern further by cloning two *PIN* promoters and fusing these with the GUS reporter gene.

## Materials and methods

### Identification of StPIN gene sequences

The potato *PIN* sequences were retrieved by blasting all identified *A. thaliana PIN* gene sequences against the potato genome sequence (Table S1). Identified Potato PIN sequences were aligned (Crystal W2 alignment, http://www.ebi.ac.uk/Tools/msa/clustalw2/) with the *A. thaliana* PIN genes and were screened for the presence of the conserved PIN hydrophobic/hydrophilic structure. The phylogenetic tree was constructed using Megalign (DNASTAR Lazergene 9 core suite) using a Clustal methodology. Prediction of the location of the trans-membrane domains within the protein sequence was performed with TMHMM program (v2) (http://www.cbs.dtu.dk/services/TMHMM/). The predicted intron-exon structure of the *StPINs* genes data was retrieved from the potato genomics browser (http://solanaceae.plantbiology.msu.edu) The *StPIN* genes were named according to sequence similarity to the *A. thaliana PIN* genes.

### Cloning and analysis of STPIN4 and StPIN2 promoter sequences

The promoter regions of *STPIN4* (2741 bp) and *StPIN2* (2983 bp) were cloned from *Solanum tuberosum* group Andigenum using Gateway technology (Invitrogen Europe BV, Blijswijk, NL). All primers used are provided in Table S2. Vector pKGFS7, harboring the GUS reporter protein was used for transformation and promoter expression studies (Karimi et al., 2002). Transgenic plants harboring the prom*StPIN2*::GUS and prom*StPIN4*::GUS construct were obtained by *Agrobacterium*-mediated transformation (*AGL0*) of *S. tuberosum group* Andigenum *in vitro* plantlets as described previously (Visser et al., 1989). The independent transformed clones were clonally propagated *in vitro* four times prior to greenhouse experiments. The GUS staining assays was done as described previously (Stomp, 1992). The incubation of the tissues in GUS substrate X-Gluc was performed overnight at 37°C. The tissues were washed once with 70% ethanol prior to imaging.

### Expression analysis of StPIN genes

*Solanum tuberosum* group Andigenum plants were grown in the greenhouse and transferred to “short day” conditions when the plants were at the 6th fully expanded leaf stage. Stolon tips were harvested under long days conditions (day 0; LD 16 h light) after which plants were transferred to “short days” conditions (SD 8 h light), and harvested at day 2, 4, 6, and 8 after the switch to SD. Total RNA was extracted using the QiagenRNaesy Plant mini kit (QIAGEN Benelux B.V.Venlo, NL) and DNase I treated (Invitrogen). 1μg of RNA was used for cDNA synthesis and the final product was diluted 20 times in a total volume of 400 ul (BioradiScriptcDNA synthesis kit, Bio-Rad Laboratories B.V., Veenendaal, NL). qRT-PCR was performed using the BioradiQ™ SYBR® Green Supermix on a Bio-Rad cycler. The reactions were performed in triplicate in a final volume of 10 μl, containing 5 μl of SYBR® Green Supermix, 100 nM of each primer, PCR-grade water and 2 μl of cDNA sample. Reactions were incubated at 95°C for 5 min, followed by 40 cycles at 95°C for 15 s and 60°C for 1 min. *eIF3e* was used as a reference gene (all qRT-PCR primer sequences are provided in Table S3). RNA-seq data of various tissues of the RH89-039-16 genotype (referred to as RH) was retrieved from the potato genomic browser and includes flower, leaves, shoot apex, stem stolon, young tuber, and root tissue. For each tissue, RNA-seq reads are mapped against the predicted gene structures indicating relative expression levels (nr of fragments per kb per million reads or FPKM).

## Results and discussion

### Identification of the PIN gene family members in potato

**Figure 1:**
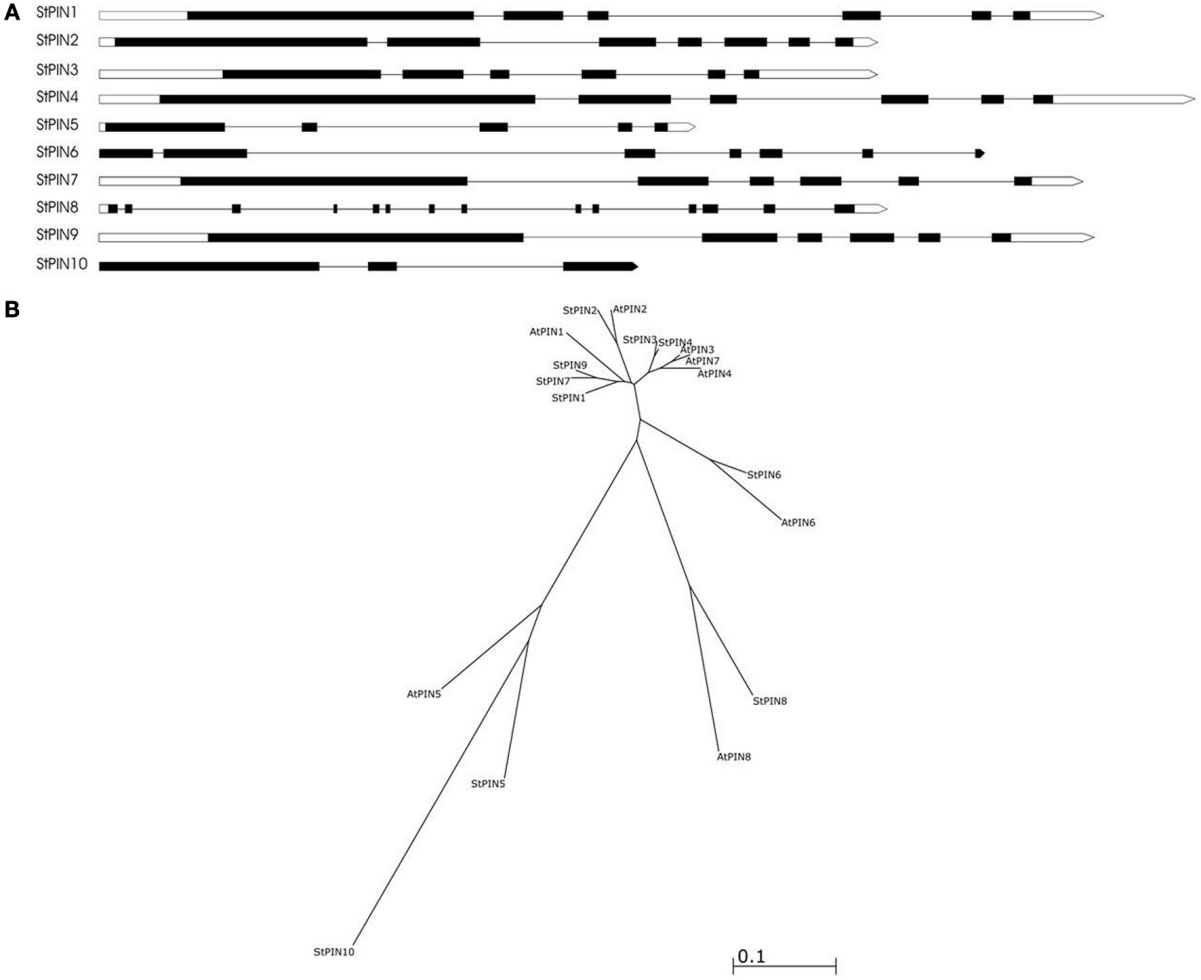
**The predicted gene structure and phylogenetic relationships of the StPIN family of genes**. **(A)** The direction of transcription is from left to right. Black boxes represent the exons, white boxes represent the 5' and 3' UTR and the lines represent the introns. **(B)** Phylogenetic tree of the PIN proteins of *A. thaliana* and *S. tuberosum*. Accession numbers of the *A. thaliana* sequences are given in Table S4.

**Figure 2:**
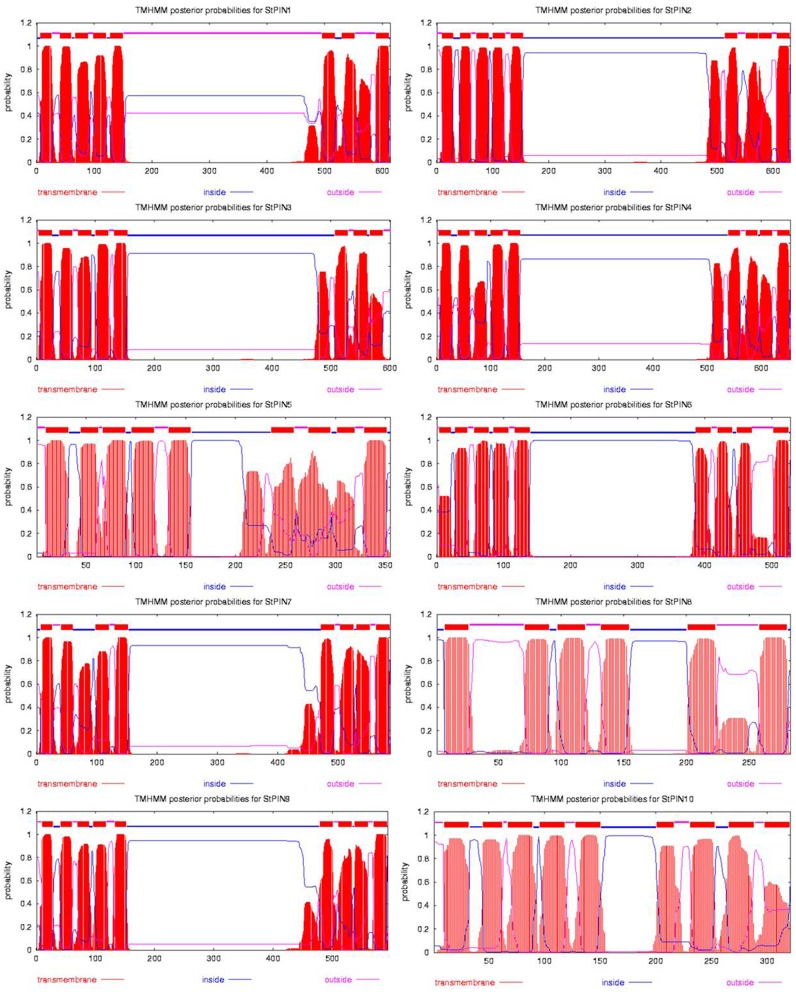
**Hydropathy analysis of the 10 StPIN proteins found in the potato genome**. Red peaks and blocks represent the trans-membrane domains, pink lines the areas of the proteins predicted to be outside the cell, and the blue lines represent the areas of the proteins predicted to be inside the cell.

Based on sequence similarity with *A. thaliana* PIN proteins and the presence of trans-membrane domains, a total of ten potato *PIN* genes could be identified using the potato reference genome sequence (Table S1). As in *A. thaliana*, the potato *PIN* genes have been divided in two groups based on the length of their protein-coding region, the long *PINs* and the short *PINs*. The group of the short StPIN proteins comprises three members (StPIN10; 321 amino acid residues, StPIN5; 355 amino acid residues, and StPIN8; 358 amino acid residues); the same number of members as the *A. thaliana* group and one less than the tomato group (Pattison and Catalá, 2011). The remaining 7 StPINs that form the group of long PINs have a size range of 412–631 amino acids. The difference in length between the proteins is mainly due to the difference in length of the hydrophilic region located between the trans-membrane domains present at both ends of the protein. The N-terminus and C-terminus regions that contain the trans-membrane domains of the proteins are highly conserved in the *A. thaliana* and potato PIN proteins Table S5. For 8 out of the 10 *PIN* genes, the gene annotation predicts 5 to 7 exons. Exceptions are *StPIN8* with 14 exons and *StPIN10* with 3 exons, two of the three short *StPINs* (Figure 1A). At the N-terminus of the proteins, all StPINs have 4 to 5 trans-membrane domains followed by the hydrophilic loop that varies in length. The C-terminus end has 3 to 4 trans-membrane domains, with the exception of StPIN8 that has two trans-membrane domains (Figure 2).

The phylogenetic analysis reveals the evolutionary relationship between the AtPIN and StPIN predicted PIN proteins (Figure 1B). (Alignment of the StPIN and AtPIN proteins provided in Table S5). In three cases, an *AtPIN* gene was found to group with a single *StPIN* gene (*AtPIN2* with *StPIN2, AtPIN6* with *StPIN6*, and *AtPIN8* with *StPIN8*). In contrast, *AtPIN1* clusters together with potato *StPIN1, StPIN7* and *StPIN9*, while *AtPIN3, 4*, and *7* form a group with *StPIN3* and *StPIN4* (Accession numbers of the *A. thaliana* PIN genes used in alignment provided in Table S4). The short *AtPIN5* is located in a branch with *StPIN5* and *StPIN10*. These results indicate the evolutionary paths that resulted in the *PIN* family in *A. thaliana* and in potato. It seems likely that *AtPIN1* shares a common ancestor with *StPIN1, StPIN7*, and *StPIN9*. The fact that *StPIN7* and *StPIN9* are very similar in sequence level and are located on the same chromosome indicates a recent duplication event. In addition, the fact that *AtPIN3, 7*, and *4* are located on the same branch indicates a common ancestor with *StPIN4* and *StPIN3* that are also located on the same branch. Clustering of proteins based on sequence similarity between *AtPIN* and *StPIN* genes implies similar functional roles or sub-functionalization in species-dependent developmental processes.

### Tissue-specific expression of potato PIN gene family members

**Figure 3:**
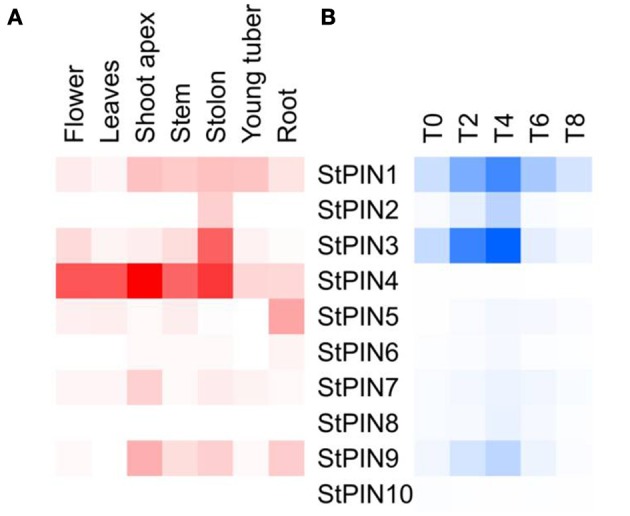
**Heat map of expression of the StPIN genes in the corresponding tissues according to the RNA-seq data of RH and in the tuber developmental series (time points T0 to T8)**. In **(A)**, expression levels in the various tissues are indicated by shades of red, where white indicates no expression. In **(B)**, for stages T0 to T8 of the developmental series, 0 to 8 days after induction to tuberise, shades of blue represent fold increase in the expression of the corresponding gene and white indicates the lowest expression detected. Lowest expression is detected for StPIN10 at stage T4 (*C*(*t*) = 36.29), and highest expression is detected for StPIN1 at stage T8 (*C*(*t*) = 25.14).

Together with the potato genome sequence, additional data including RNA-seq-generated expression data became available, targeting a number of different potato tissues or developmental stages. Based on potato genotype RH RNA-seq tissue libraries, we built a heat-map that shows sites of *StPINs* expression throughout the plant (Figure 3A). Only *StPIN4* and *StPIN1* are expressed in all tissues, with *StPIN4* expressed at a high level while all remaining potato *PIN* genes seem to have some degree of tissue specificity. *StPIN2* is expressed predominantly in the stolon tissue, and *StPIN3* is present at a low level in all tissues with the exception of roots. *StPIN5* is predominantly expressed in the roots.

In each tissue, a different set of *StPINs* is expressed (Figure 3A). In the roots *StPIN1, 4, 5*, and *9* gene expression was detected, while in the stolon *StPIN3* and *StPIN4* are predominantly expressed while *StPIN1, 2*, and *9* are present at low levels. In the young tuber, transcript from 5 out of 10 *StPIN* genes was detected, with StPIN1 and StPIN4 exhibiting the highest expression levels. In flowers 6 *StPINs* were expressed, but only *StPIN1, StPIN4*, and *StPIN3* showed relatively strong expression. In the shoot apex, stem and roots 5 *StPINs* (*StPIN1*, 3, *4, 7, 9)* were expressed, however, the expression level of *StPIN4* was the highest in the apex. Tissue-specific expression of several *StPIN* genes points out to possible functional redundancy among the *PIN* genes.

The PAT mediates translocation of auxin from the sites of biosynthesis to the lower parts of the plant and the roots. Little knowledge exists on the fate of auxin after it reaches the root tips and enters the outer epidermis cells. *StPIN5* is predominantly expressed in the root, which is interesting as the *A. thaliana* ortholog (*AtPIN5*) has been shown to participate in the subcellular localization and homeostasis of auxin (Mravec et al., 2009). Based on sequence similarity between *AtPIN5* and *StPIN5*, it is likely that StPIN5 is involved in auxin homeostasis in potato. However, the functional role for *StPIN5* in auxin homeostasis and subcellular localization in the roots needs to be confirmed.

The stolon is an underground stem that grows diageotropically. The *A. thaliana PIN2* is known to have a role in the gravitropic response in roots by directing auxin flow to one side of the root and thus mediating differential growth across both sides of the root (Luschnig et al., 1998; Muller et al., 1998). In a similar manner, light affects distribution of AtPIN3 protein, that regulates auxin distribution and differential growth in response to light (Okada et al., 1991). As a result, the comparison between the *StPINs* expressed in stem and stolon becomes intriguing, as stolons are generally deprived of light. StPIN2 protein seems to be highly homologous to AtPIN2 (72% similarity at the amino acid level). In potato, *StPIN2* is expressed in the stolon but not at high levels the stem, and this may be accounted for differential gravitropic response of the stolon in comparison to the aerial shoot. Our results provide good insights into diageotropic growth response of the stolon in comparison to the aerial shoot growth and the potential involvement of PIN proteins in this process.

### PIN expression during potato tuber development

In potato, previous research on tuber development revealed two *StPINs* (later named StPIN4 and StPIN2) that have a peak in expression 4 days after tuber induction, indicating a role for auxin in the development of the potato tuber (Kloosterman et al., 2008). In order to investigate the expression profile of the *PIN* family of genes in the early stages of tuber induction, we performed qRT-PCR on stolon tip samples collected 0, 2, 4, 6, and 8 days (stages T0–8, respectively) after plants were transferred to short days to induce tuberization (Figure 3B). The results revealed that several *StPINs* are highly expressed in the swelling stolon (stage T8). In addition, a peak in expression levels is noticed for six *PIN* genes at (*StPIN1, 2, 3, 7, 8*, and *9*) four days after tuber induction, ranging from 2 to 10-fold increase in comparison to T0. By stage T8 (day 8), the expression levels of *StPIN1, StPIN6*, and *StPIN8* were the same as at stage T0, but a 2 to 5-fold decrease was observed for *StPIN3, 6*, and *9*. The expression levels of two more *StPINs* (*StPIN4, 10*) remain relatively stable in all stages, while *StPIN7* and *StPIN5* show a gradual increase of expression. Auxin has been shown to be a positive regulator of *PIN* gene expression (Vieten et al., 2005). The up-regulated profile of six *StPIN* genes at T4 is in accordance with our previous findings where auxin was shown to accumulate in the potato stolon tip after tuber induction and prior to first visible swelling (day 8) (Roumeliotis et al., 2012). Auxin is known to participate in many developmental events, such as embryogenesis, flower development, lateral root formation, and tuber initiation where it has been associated with the establishment of meristem identity (Marchant et al., 2002; Krizek, 2011; Luo et al., 2011; Roumeliotis et al., 2012). This peak in expression is probably important to distribute auxin to the correct sites where it may be required in the formation of a new organ, the tuber.

The RNA-seq data obtained from tubers and the qRT-PCR expressions of *PIN* genes during tuber development are not directly comparable, as sampling was done on different genotypes, time courses and tissue types. Nevertheless, *StPIN* genes that are predominantly expressed in the stolon tissue are also expressed in the stolon developmental stages, with the exception of *StPIN4*. In the tissue panel, *StPIN4* seems to be the predominant *PIN* gene expressed in the stolon as well as in almost all other tissues. Surprisingly, in the stolon developmental series the *StPIN4* expression is lower compared to the other *StPIN* genes. It is possible that *StPIN4* is “down regulated” once the potato plant is induced to tuberize, as shown by the lower *StPIN4* expression in the tuber. All other *PIN*s found to be expressed in the stolon tissue in the RNA-seq data, such as *StPIN1, 2, 3*, and *9* are also detected in the RT-PCR data. The compartmental distribution of auxin adjacent to the vascular system in the stolon and swelling tuber may be a result of the combined expression of all the *PIN* genes during early stages of tuberization (Roumeliotis et al., 2012).

### StPIN2/4 promoter GUS staining

**Figure 4:**
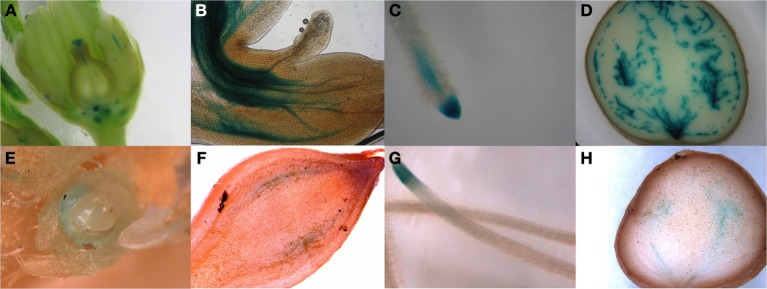
**Pin promoter expression study**. Gus expression driven by the StPIN4 promoter is detected in flowers **(A)**, stolon tip **(B)**, root tip **(C)** swelling tuber **(D)** and StPIN2 expression was found the in flower bud **(E)**, swelling stolon **(F)**, root tip **(G)** and in swelling tuber **(H)**.

In order to identify the regions of expression of the *StPIN* genes in stolons and in young tubers in more detail, the promoters of two *StPIN* genes were cloned in front of a GUS reporter gene referred to as *promStPIN2::GUS* and *promStPIN4::GUS*. In transgenic plants harboring the *promStPIN4::GUS* construct, GUS staining was detected in flowers, stolon tips, root tips, and swelling tubers (Figures 4A–D). More specifically, GUS staining was visible in the stigma and the style of flowers and in the stem just below the ovary and petals (Figure 4A). In stolon tissue, GUS staining was detected in the vascular tissue (Figure 4B) in the sub apical region where the swelling of the stolon takes place, while in roots GUS staining was restricted to the root tip (Figure 4C). In tubers, GUS staining was visible in the vascular system, and in the perimedullary region (Figure 4D). GUS staining was also detected in the basal part of the pith close to the heel of the tuber where it attaches the stolon. In the transgenic plants carrying the *promStPIN2::GUS* construct, GUS staining was also detected in flower buds, roots, swelling stolons, and young tubers (Figures 4E–H). In mature flowers, no GUS staining was detected. In addition, stolon tips did not have any GUS staining, until the stolon tip started to swell (first visible sign of tuberization), with the staining being restricted to the vascular tissues (Figure 4F). In the mature tuber (Figure 4H), GUS staining was observed in the pith and in the perimedullary region, partially overlapping with the *StPIN4* promoter driven GUS staining. In some tissues such as the young tuber, *StPIN4* promoter and *StPIN2* promoter GUS staining is overlapping in the same regions in the vascular tissue, in the pith and in the perimedullary region. A large portion of the tuber growth is attributed to the thickening of the perimedullary region therefore distribution of auxin in this tissue by the PIN proteins can be of importance (Xu et al., 1998). It is also interesting to point out that tuberization *in vitro* does not produce full size tubers, due to the fact that cell divisions stop when the young tuber reaches a size of 0.8 cm in diameter. Investigating the auxin content and auxin distribution in *in vitro* tuberization experiments could help us elucidate if auxin homeostasis is important for achieving full tuber growth. In contrast, in flowers and root tips, GUS staining driven by the *StPIN4* and *StPIN2* promoters seems to have a different spatial expression patterns. In roots, *StPIN4* promoter driven GUS staining is observed primarily in the root cap and in the stele, while *StPIN2* promoter GUS staining is observed in the apical meristem adjacent to the root cap, close to the elongation zone. It seems that the two *PIN* genes might have partially overlapping roles in distributing auxin in some tissues, and unique functionality in others. Redundancy between different members of the PIN family has been reported earlier in tomato (Pattison and Catalá, 2011). The overlap of expression of the *StPINs*, as suggested also by the expression heatmap (Figure 3A) in the various tissues implies that *StPINs* in potato might act synergistically to direct plant growth and development.

## Conclusions

In this study, we identified the PIN family of proteins in potato and discuss their putative roles based on the homology to the *A. thaliana* PIN proteins. Using the potato genome sequence, we have identified ten potato *PIN* gene family members and studied their relative expression levels in various tissues and during early stages in tuberization. Promoter analysis of the two potato *PIN* homologs revealed the sites of expression in aerial parts of the plant, as well as in the stolons during the very first days after tuber induction. Based on these results and what is known about the changes in auxin content during early stages of tuber development, we discuss a possible role for StPIN proteins in redistributing auxin in the swelling stolon and developing tuber.

### Conflict of interest statement

The authors declare that the research was conducted in the absence of any commercial or financial relationships that could be construed as a potential conflict of interest.

## Supplementary material

Table S1 **Genomic location of the StPIN genes**.

Table S2 **Primers used for cloning**.

Table S3 **Primers for qRT PCR used in this study**.

Table S4 **Accession numbers of the *A. thaliana* PIN genes used in alignment**.

Table S5 **Alignment of the StPIN and AtPIN proteins was performed with ClustalW2- Multiple Sequence Alignment (http://www.ebi.ac.uk/Tools/msa/clustalw2/), alignment type: slow, and alignment options: default (“^*^”, identical; “:”, conserved substitutions; “.”, semi-conserved substitution)**.
