# Supplementary material for: The PIN family of proteins in potato and their putative role in tuberization
Source: Front Plant Sci. 2013 Dec 19;4:524. doi: 10.3389/fpls.2013.00524 (PMC3867687; doi:10.3389/fpls.2013.00524)
Supplement: Table S1 — Genomic location of the StPIN genes. [file DataSheet1.PDF]

Supplementary Table 1. Genomic location of the *StPIN* genes.

PIN gene	Genomic area	PGSC transcript number
StPIN1	PGSC0003DMB000000026:1618465..1622012	PGSC0003DMT200014752
StPIN2	PGSC0003DMB000000613:194621..197391	PGSC0003DMT200048251
StPIN3	PGSC0003DMB000000322:55773..58731	PGSC0003DMT200015267
StPIN4	PGSC0003DMB000000051:1583809..1580797	PGSC0003DMT200078330
StPIN5	PGSC0003DMB000000068:6791..3618	PGSC0003DMT200046253
StPIN6	PGSC0003DMB000000227:699831..693452	PGSC0003DMT200079013
StPIN7	PGSC0003DMB000000008:4038046..4041324	PGSC0003DMT200072459
StPIN8	PGSC0003DMB000000004:2324342..2326593	PGSC0003DMT200003570
StPIN9	PGSC0003DMB000000379:549866..546988	PGSC0003DMT200021600
StPIN10	PGSC0003DMB000000123:720022..721629	PGSC0003DMT200027309
