# Supplementary material for: The PIN family of proteins in potato and their putative role in tuberization
Source: Front Plant Sci. 2013 Dec 19;4:524. doi: 10.3389/fpls.2013.00524 (PMC3867687; doi:10.3389/fpls.2013.00524)
Supplement: Table S2 — Primers used for cloning. [file DataSheet2.PDF]

Supplementary Table 2. Primers used for cloning.

StPIN2 prom	F	CACCgtcacatcctcagaccaag
	R	tcaaatttcgagaatccaacaa
StPIN2 gene	F	CACCatgcacaactaaatgagtatga
	R	aaaagagagaaaagtatgaaag
StPIN4 prom	F	CACCaaagttccaagttcaatc
	R	tttcgcaaaaaaaattgtcaaatag
StPIN4 gene	F	CACCaaaatgatcacttggcacga
	R	caatggaggccttcaaaaa
