# Supplementary material for: The PIN family of proteins in potato and their putative role in tuberization
Source: Front Plant Sci. 2013 Dec 19;4:524. doi: 10.3389/fpls.2013.00524 (PMC3867687; doi:10.3389/fpls.2013.00524)
Supplement: Table S3 — Primers for qRT PCR used in this study. [file DataSheet3.PDF]

Supplementary Table 3. Primers for qRT PCR used in this study.

**Primer Name**	**sequence** (in 5'----> 3' order)
StPIN1 F RT1	AGGCAGCTCTACCACAAGGA
StPIN1 R RT1	CAAGCCCAACAAACAAAACC
StPIN2 F RT2	TCATCTAAAGGGCCAACACC
StPIN2 R RT2	GTTGTATAGCTCCCCGCTCA
StPIN3F RT2	ACCAATGCTCAGGGATCAAC
StPIN3R RT2	GTCAGCTGCAATGAATCGAA
StPIN4 F RT2	GGGACCCACTGGACTGACTA
StPIN4 R RT2	ACTTGCTGGAGGCATCTGTT
StPIN5F RT1	CCCCAAGCAGTGACATCTTT
StPIN5R RT1	CGAAATTATGGTGCCAACAA
StPIN6F RT2	GAATCCGCATTTTCATCCTC
StPIN6R RT2	CCCGTTATGTAAAGGCGTGT
StPIN7 F RT1	CAGCCGAGCTGTTTCCTAAC
StPIN7 R RT1	TTTTCGCCACACCATAATCA
StPIN8F RT2	ATAGCATGCGGAACCAAAAA
StPIN8R RT2	TGTCCCCTTAGTCCAACAGC
StPIN9 F RT2	TTGGCCTAACTTGGTCCTTG
StPIN9 R RT2	AAACATTGCCATTCCGAGAC
StPIN10 F RT1	ATGTCAAAGGCAGGAAGTGG
StPIN10 R RT1	GTGCCACCAGCCATTACTTT
eIF3e F	GGAGCACAGGAGGAAGATGAAGGAG
eIF3e R	CGTTGGTGAATGCGGCAGGAAGGAG
