# Supplementary material for: The PIN family of proteins in potato and their putative role in tuberization
Source: Front Plant Sci. 2013 Dec 19;4:524. doi: 10.3389/fpls.2013.00524 (PMC3867687; doi:10.3389/fpls.2013.00524)
Supplement: Table S4 — Accession numbers of the A. thaliana PIN genes used in alignment. [file DataSheet4.PDF]

Supplementary Table 4.Accession numbers of the *A. thaliana PIN* genes used in alignment.

Arabidopsis	Accession number
AtPIN1	AF089084
AtPIN2	AF086906
AtPIN3	AF087818
AtPIN4	NM_126203
AtPIN5	NM_121659
AtPIN6	NM_121659
AtPIN7	NM_102156
AtPIN8	NM_121514
