# Supplementary material for: The PIN family of proteins in potato and their putative role in tuberization
Source: Front Plant Sci. 2013 Dec 19;4:524. doi: 10.3389/fpls.2013.00524 (PMC3867687; doi:10.3389/fpls.2013.00524)
Supplement: Table S5 — Alignment of the StPIN and AtPIN proteins was performed with ClustalW2- Multiple Sequence Alignment (http://www.ebi.ac.uk/Tools/msa/clustalw2/), alignment type: slow, and alignment options: default (“*”, identical; “:”, conserved substitutions; “.”, semi-conserved substitution). [file DataSheet5.PDF]

Supplementary Table 5**.** Alignment of the StPIN and AtPIN proteins was performed with ClustalW2-Multiple Sequence Alignment (http://www.ebi.ac.uk/Tools/msa/clustalw2/) , alignment type: slow, and alignment options: default ("*": identical, ":": conserved substitutions, ".": semi-conserved substitution).

```
StPIN1          MITLSDFYHVMTAVVPLYVAMILAYGSVKWWKIFSPDQCSGINRFVALFAVPLLSFHFIA 60
AtPIN1          MITAADFYHVMTAMVPLYVAMILAYGSVKWWKIFTPDQCSGINRFVALFAVPLLSFHFIA 60
StPIN7          MITVSDLYHVLTAVVPLYVAMILAYGSVKWWKIFSPDQCSGINRFVALFAVPLLSFHFIA 60
StPIN9          MISLSDLYHVLTAVVPLYVAMILAYGSVKWWKIFSPDQCSGINRFVALFAVPLLSFHFIA 60
StPIN3          MISWHDLYVVLTAVVPLYVAMILAYGSVRWWKIFSPDQCSGINRFVAIFAVPLLSFHFIS 60
StPIN4          MITWHDLYVVLTAVVPLYVAMILAYGSVRWWKIFSPDQCSGINRFVAIFAVPLLSFHFIA 60
AtPIN3          MISWHDLYTVLTAVIPLYVAMILAYGSVRWWKIFSPDQCSGINRFVAIFAVPLLSFHFIS 60
AtPIN7          MITWHDLYTVLTAVIPLYVAMILAYGSVRWWKIFSPDQCSGINRFVAIFAVPLLSFHFIS 60
AtPIN4          MITWHDLYTVLTAVVPLYVAMILAYGSVQWWKIFSPDQCSGINRFVAIFAVPLLSFHFIS 60
StPIN2          MINGKDIYDVLAAIIPLYVAMILAYGSVRWWKIFTPDQCSGINRFVAVFAVPLLGFHFIS 60
AtPIN2          MITGKDMYDVLAAMVPLYVAMILAYGSVRWWGIFTPDQCSGINRFVAVFAVPLLSFHFIS 60
StPIN8          MISLRDVYHVVAATIPLYVVMILAYISVRWGKLFSPEQCSGINKFVAKFSIPLLSFQVIS 60
AtPIN8          MISWLDIYHVVSATVPLYVSMTLGFLSARHLKLFSPEQCAGINKFVAKFSIPLLSFQIIS 60
StPIN6          -------------MVPLYFAMIVAYGSVKWWKIFSPEQCSGINRFVAVFAVPVLSFHFIS 47
AtPIN6          ------------------------------------------------------------
StPIN5          MIGWDDIYKVVVGMMPLYVALILGYGSVKWWHMFKPEQCDTINRFNCFFILPFFNFQFIA 60
StPIN10         MIEWLDIYKVIEAMMPLYLALGLGYGSVKWWHKLSAEHCDAINRLNYFFVLPFFTFDFIS 60
AtPIN5          MINCGDVYKVIEAMVPLYVALILGYGSVKWWHIFTRDQCDAINRLVCYFTLPLFTIEFTA 60


StPIN1          ANNPYTMNIRFIAADTLQKLIVLGVLAVWANVSK-----RGSLEWSITLFSLSTLPNTLV 115
AtPIN1          ANNPYAMNLRFLAADSLQKVIVLSLLFLWCKLSR-----NGSLDWTITLFSLSTLPNTLV 115
StPIN7          SNNPYAMNYRFIAADTLQKVIVLVVLAIWSRISS-----RGSLEWSITLFSLSTLPNTLV 115
StPIN9          SNNPYAMNYRFIAADTLQKIIVLFVLAIWSRVSS-----RGSLEWSITLFSLSTLPNTLV 115
StPIN3          MNNPYEMNFRFIAADTLQKVIMLIVLCIWSNVTK-----NGSLEWSITIFSLSTLPNTLV 115
StPIN4          MNNPYEMNFRFIAADSLQKVIMLVVLSLWANLTK-----NGSLEWSITIFSLSTLPNTLV 115
AtPIN3          TNNPYAMNLRFIAADTLQKIIMLSLLVLWANFTR-----SGSLEWSITIFSLSTLPNTLV 115
AtPIN7          SNNPYAMNLRFIAADTLQKLIMLTLLIIWANFTR-----SGSLEWSITIFSLSTLPNTLV 115
AtPIN4          TNDPYAMNFRFVAADTLQKIIMLVLLALWANLTK-----NGSLEWMITIFSLSTLPNTLV 115
StPIN2          TNDPYSMNYHFIAADSLQKVVILFALFIWHVFSK-----RGSLEWVITLFSLSTLPNTLV 115
AtPIN2          SNDPYAMNYHFLAADSLQKVVILAALFLWQAFSR-----RGSLEWMITLFSLSTLPNTLV 115
StPIN8          GSNLYKVNLKLLLADFIQKFLAVFLLAIFAKLKP-----KGNLTWIITGLSVSTLPNTLI 115
AtPIN8          ENNPFKMSPKLILSDILQKFLVVVVLAMVLRFWHPTGGRGGKLGWVITGLSISVLPNTLI 120
StPIN6          QNNPYQMDTKFILADTLSKILVLVLLSVWAICK-------GQLDWLITLFSVSTLPNTLV 100
AtPIN6          ----------FILADTLSKIFVFVLLSLWAVFFK-----AGGLDWLITLFSIATLPNTLV 45
StPIN5          NINPYNLNYLFLTGDVIAKALVILILVLWANFYR-----KGSFSWGITTFSLSTLNNTLV 115
StPIN10         QVNPYKMNYPFICGDLIAKAIIGFFLTLWANFYS-----KGNFSWSITTFSFCSLTNALV 115
AtPIN5          HVDPFNMNYRFIAADVLSKVIIVTVLALWAKYSN-----KGSYCWSITSFSLCTLTNSLV 115
                          :: .* : * .    * :            *   * ** :*.. * *:*:

StPIN1          MGIPLLKGMYGEFSGSLMVQIVVLQCIIWYTLMLFMFEFRGARLLISEQFP-DTAGSIVS 174
AtPIN1          MGIPLLKGMYGNFSGDLMVQIVVLQCIIWYILMLFLFEYRGAKLLISEQFP-DTAGSIVS 174
StPIN7          MGIPLLKGMYGDASGSLMVQIVVLQCIIWYTLMLFLFEYRGARMLIAEQFP-DTGGSIVS 174
StPIN9          MGIPLLKGMYGDASGSLMVQIVVLQCIIWYTLMLFLFEYRGARMLIAEQFP-DTGGSIIS 174
StPIN3          MGIPLLIAMYGEYSGSLMVQVVVLQCIIWYTLLLFLFEFRGAKMLIMEQFP-ETAGEIVS 174
StPIN4          MGIPLLIAMYGEYSGSLMVQVVVLQCIIWYTLLLFLFEYRGAKMLIMEQFP-ETAASIVS 174
AtPIN3          MGIPLLIAMYGEYSGSLMVQIVVLQCIIWYTLLLFLFEFRGAKMLIMEQFP-ETAASIVS 174
AtPIN7          MGIPLLIAMYGEYSGSLMVQIVVLQCIIWYTLLLFLFEYRGAKILIMEQFP-ETGASIVS 174
AtPIN4          MGIPLLIAMYGTYAGSLMVQVVVLQCIIWYTLLLFLFEYRGAKLLIMEQFP-ETGASIVS 174
StPIN2          MGIPLLKAMYGDFSGNLMVQIVVMQSVIWYTLMLFMFEYRGAKLLIGEQFP-ETAASITS 174
AtPIN2          MGIPLLRAMYGDFSGNLMVQIVVLQSIIWYTLMLFLFEFRGAKLLISEQFP-ETAGSITS 174
StPIN8          LGIPLIKAIFGDAAAELLAQLIALQSLVWYNLLLLLFELN-----ATKES------YMMS 164
AtPIN8          LGMPILSAIYGDEAASILEQIVVLQSLIWYTILLFLFELN-----AARALP-SSGASLEH 174
StPIN6          MGIPLLNAMYGDFTQSLMVQLVVLQCIIWYTLLLFLFEYRAATILIKNQFPGNVAASITK 160
AtPIN6          MGIPLLQAMYGDYTQTLMVQLVVLQCIIWYTLLLFLFELRAARLLIRAEFPGQAAGSIAK 105
StPIN5          VGVPLMKAMYGDLGVDLVVQAAVIQALLWLTSLLFALEFWKTKMNNNINGI--------- 166
StPIN10         MGIPVMNAMSPQVGVDLVIQSLAIQFLIWSIIIQFMMELKNAKDEIMACEG--------- 166
AtPIN5          VGVPLAKAMYGQQAVDLVVQSSVFQAIVWLTLLLFVLEFRKAGFSSNNISD--------- 166
                :*:*:  .:       :: *  .:* ::*   : : :*


StPIN1          IHVDSDVMSLDGRQVLETEAELKEDGKLHVTVRKSNASRSDIFSRRS-----GGFSSTTP 229
AtPIN1          IHVDSDIMSLDGRQPLETEAEIKEDGKLHVTVRRSNASRSDIYSRRS-----QGLS-ATP 228
StPIN7          FKIDSDVISLDGKEPLETQAEVGDDGKLHVVVRKSTSSRSEIFSRMSH-GHNTGGLSMTP 233
StPIN9          FKVDSDIISLDGKEPLETQAEVGDDGKLHVTVRKSTSSRSEIFSRRSH-GPNSG-LSLTP 232
StPIN3          FKVESDVVSLDGQDFLETDAELGQDGKLHVTVRKSNVSRRSFAM--------------TP 220
StPIN4          FKVESDVVSLDGHDFLETDAEIGQDGKLHVTVRKSNASRRSFAM--------------DH 220
AtPIN3          FKVESDVVSLDGHDFLETDAEIGDDGKLHVTVRKSNASRRSFCG-----------PNMTP 223
AtPIN7          FKVESDVVSLDGHDFLETDAQIGDDGKLHVTVRKSNASRRSFYGGGG--------TNMTP 226
AtPIN4          FKVESDVVSLDGHDFLETDAEIGNDGKLHVTVRKSNASRRSLMM--------------TP 220
StPIN2          FRVESDVISLNGREPLQADAEIGDDGKLHVIVRRS-SASSIISSYNK---GILQSNNMTP 230
AtPIN2          FRVDSDVISLNGREPLQTDAEIGDDGKLHVVVRRSSAASSMISSFNKSHGGGLNSSMITP 234
StPIN8          PSEVAVELEVPGEPELEKD----------------------------------------- 183
AtPIN8          TGNDQEEANIEDEPKEEEDEEE-------------------------------------- 196
StPIN6          FEIDNDVISLDGRNPLCTESEINGNGRIHVRIRRSTSSAPESAFSSS--------IGITP 212
AtPIN6          IQVDDDVISLDGMDPLRTETETDVNGRIRLRIRRSVSSVPDSVMSPS--------LCLTP 157
```

```
StPIN5          ANNNSVELGNINTTTQM------------------------------------------- 183
StPIN10         ANQDLEGNDNNNAS---------------------------------------------- 180
AtPIN5          VQVDNINIESGKRE---------------------------------------------- 180


StPIN1          RPSNLTNAEIYSLQSSRNP-TPRGSSFNHTDFYSMVAGTAGRNSNFG-ANDVYGMSN--- 284
AtPIN1          RPSNLTNAEIYSLQSSRNP-TPRGSSFNHTDFYSMMASGGGRNSNFGPGEAVFG------ 281
StPIN7          RPSNLSNTEIYSLQSSRNM-TPRDSNFNHNDIYSMVNGKNN--ATMSPRTSNFG------ 284
StPIN9          RPSNLTNAEIYSLQSSRNP-TPRGSSFNHTDFYSMVNGKN---ANMSPRNSNFG------ 282
StPIN3          RPSNLTGAEIYSLNSSRNP-TPRGSNFNHTDFYAMMGFPG-RLSNFGPADS--------- 269
StPIN4          RPSNLTGAEIYSLSSSRNP-TPRGSNFNHNDFYSMMGFPGGRLSNFGPADMYSVQS---- 275
AtPIN3          RPSNLTGAEIYSLST-----TPRGSNFNHSDFYNMMGFPGGRLSNFGPADMYSVQS---- 274
AtPIN7          RPSNLTGAEIYSLNT-----TPRGSNFNHSDFYSMMGFPGGRLSNFGPADMYSVQS---- 277
AtPIN4          RPSNLTGAEIYSLSS-----TPRGSNFNHSDFYSVMGFPGGRLSNFGPADLYSVQS---- 271
StPIN2          RASNLTGVEIYSVQSSREP-TPRASSFNQSDFYAMF-ASKTASPKHGYTNSYG------- 281
AtPIN2          RASNLTGVEIYSVQSSREP-TPRASSFNQTDFYAMFNASKAPSPRHGYTNSYGGAGAGPG 293
StPIN8          ------------------------------------------------------------
AtPIN8          ------------------------------------------------------------
StPIN6          RASNLSNAEIFSVHTP---------------LHNG--DIPFGHGDLG-VGFR-------- 246
AtPIN6          RASNLSNAEIFSVNTPNNRFFHGGGGSGTLQFYNGSNEIMFCNGDLGGFGFTRPGL---- 213
StPIN5          ------------------------------------------------------------
StPIN10         ------------------------------------------------------------
AtPIN5          ------------------------------------------------------------


StPIN1          -------NSRGPTPRPSNYEEESGK---SRFNNYLGGAAAPAPTQSNSN-------TNYP 327
AtPIN1          --------SKGPTPRPSNYEEDGGP---AKP-TAAGTAAGAGRFHYQSGGSGGGGGAHYP 329
StPIN7          ---------------NLGFDEES------GFGKTN---------------------VGYP 302
StPIN9          ---------------NYGHDEESGV---AGFGRGNGVYGQGN--------------AGYP 310
StPIN3          ------------TPRPSNFEENCTQGALT-SSPKFGFYPAQS---------------NYP 301
StPIN4          --------SRGPTPRPSNFEENCAPGGLVQSSPRFGYFPAQQP-----------APGSYP 316
AtPIN3          --------SRGPTPRPSNFEENCA----MASSPRFGYYPGG-------------GAGSYP 309
AtPIN7          --------SRGPTPRPSNFEESCA----MASSPRFGYYPGG-------------APGSYP 312
AtPIN4          --------SRGPTPRPSNFEENNA----VKYGFYNNTNSSVP------------AAGSYP 307
StPIN2          GDVFSVQSSKGPTPRTSNFEEEMSKIGSNKKNRGGRSMSGELYNSGSNAST---NGLVYP 338
AtPIN2          GDVYSLQSSKGVTPRTSNFDEEVMKT-AKKAGRGGRSMSGELYNNNSVPS--------YP 344
StPIN8          ------------------------------------------------------------
AtPIN8          ------------------------------------------------------------
StPIN6          ---------AASTRLSGGYASSDAYS-------------------------------LQP 266
AtPIN6          ---------GASPRRLSGYASSDAYS-------------------------------LQP 233
StPIN5          ------------------------------------------------------------
StPIN10         ------------------------------------------------------------
AtPIN5          ------------------------------------------------------------


StPIN1          AP-NPGMFS----PSNNG------TKAHKNTA------KKGD--QEG-GKDLHMFVWSSS 367
AtPIN1          AP-NPGMFS----PNTGGG---GGTAAKGNAP------VVGGKRQDGNGRDLHMFVWSSS 375
StPIN7          APTNAGIFS----PVTGG----PGTKKKANG-------TESG-------KDLHMFVWSSS 340
StPIN9          APTNAGIFS----PATG-----PVTKKKANGG------TEGG-------KDLHMFVWSSS 348
StPIN3          AP-NPEIASIVPKNTKNQQ-----QLQVHSQHQHQQQQQNGKAS-HDAKELHMFVWSSS 354
StPIN4          AP-NPEISSAVPKSTKPQQPNVQTQKQEVQQQQQQQQQQPNAKANNHDAKELHMFVWSSS 375
AtPIN3          AP-NPEFSSTTTSTANKSV----NKNPKDVNTNQQTTLPTGGKSNSHDAKELHMFVWSSN 364
AtPIN7          AP-NPEFS-----TGNKTG----SKAPKENHH-------HVGKSNSNDAKELHMFVWGSN 355
AtPIN4          AP-NPEFSTG---TGVSTKP---NKIPKENQQQLQ----EKDSKASHDAKELHMFVWSSS 356
StPIN2          PP-NPMFSGQRKKEVGSGS-----GVPTPVSVPVPVPIPVPMSNSNNNSKELHMFVWSSS 392
AtPIN2          PP-NPMFTG----STSGAS-----GVKKKESGGGGSGGGVGVGGQN---KEMNMFVWSSS 391
StPIN8          ------------------------------------------------------------
AtPIN8          ------------------------------------------------------------
StPIN6          TPRGSNFNELDTITVTTSGN-------------------------------TPMWVMSPV 295
AtPIN6          TPRASNFNELD---VNGNG--------------------------------TPVWMKSPA 258
StPIN5          ------------------------------------------------------------
StPIN10         ------------------------------------------------------------
AtPIN5          ------------------------------------------------------------


StPIN1          NSPVSD----VFGGHDYTANLDQP---AAPNKDVRVPISPGKVEGQ-------------- 406
AtPIN1          ASPVSD----VFGGGGGNHHADYS---TATN-DHQKDVKISVPQGN-------------- 413
StPIN7          ASPVSEGGIHVFRGG-DFGNELGI---GHHSKDYDDFGREEFSLGD-------------- 382
StPIN9          ASPVSEGGIHVFRGGGDYGNELGV---GAHPKDYDEFGREEFTFGN-------------- 391
StPIN3          ASPVSEAAGLHVFGGTDFSANEQS---CQSDGAKEIRMLVSDHPQSGDNK--VISQD--F 407
StPIN4          NSPVSEAGGLHVFGGNDFSANEQS---GRSDGAKEIRMLVSDHTQNGDSK--AIPQTGEF 430
AtPIN3          GSPVSDRAGLNVFGGAPDNDQGGR---SDQG-AKEIRMLVPDQSHNGETKAVAHPASGDF 420
AtPIN7          GSPVSDRAGLQVDNGA--NEQVGK---SDQGGAKEIRMLISDHTQN------AGPMNGDY 404
AtPIN4          ASPVSDVFG----GGAGDNVATEQ---SEQG-AKEIRMVVSDQPRK--------SGGDDI 400
StPIN2          ASPTSEGNHKHAINRGDSSELG-----VLDASKAVLQQEIAAAREN-------------- 433
AtPIN2          ASPVSEANAKNAMTRGSSTDVSTDPKVSIPPHDNLATKAMQNLIEN-------------- 437
StPIN8          ------------------------------------------------------------
AtPIN8          ------------------------------------------------------------
StPIN6          AGKVFK------QASPSSKMAWES-----SCLNGERQGYRDDVGEK-------------- 330
AtPIN6          AGRIYR------QSSP--KMMWES-----GQRHAAKD-INGSVPEK-------------- 290
StPIN5          ------------------------------------------------------------
StPIN10         ------------------------------------------------------------
AtPIN5          ------------------------------------------------------------
```

```
StPIN1          ----------------RNNNQENYMERDDFSFANRDGADQMNNQDGEKAGENK---AKVM 447
AtPIN1          ----------------SNDNQ--YVEREEFSFGNKDDDSKVLATDGGNNISNKTTQAKVM 455
StPIN7          ----------------KNNSNGCHREEPVLKKLGSSSTAELFP-----NTAN-ETKATAM 420
StPIN9          ----------------KQNLNGNDREGPVVR---SSSTTELRP-----KIAQEETKATAM 427
StPIN3          GGEEFSFGGDVGGG-DGKNKDEKKEEKEGLTGLNTGAT-----------GVQDSGTGKQM 455
StPIN4          GGEDFTFGGANGGGKDGDEEKGEKEGPTGLTKLGSSSTSELHPKLA---GGQDAGMGKQM 487
AtPIN3          GGEQQFSF-----AGKEEEAERPKDAENGLNKLAPNSTAALQSKTGL--GGAEASQRKNM 473
AtPIN7          GGE--------------EESERVKEVPNGLHKLRCNSTAELNPKEAI--ETGETVPVKHM 448
AtPIN4          GGLDS-----------GEGEREIEKATAGLNKMGSNSTAELEAAGGD--GGGNNG--THM 445
StPIN2          -----------------ASSVSKGNVEKEIEIEDGSK---------NMEDG---EKKSQM 464
AtPIN2          -----------------MSPGRKGHVEMDQDGNNGGKSPYMGKKGSDVEDGGPGPRKQQM 480
StPIN8          ------------------------------------------------------------
AtPIN8          --------------------------------------------------------VAIV 200
StPIN6          -----------------EISFRDISNFPVQEVGAA------------DSLNTNDIIKQEM 361
AtPIN6          -----------------EISFRDALKAAPQATAAGGGASMEEGAAGKDTTPVAAIGKQEM 333
StPIN5          ---------------------------------------------------------RNI 186
StPIN10         ------------------------------------------------------------
AtPIN5          ----------------------------------------------------------TV 182

StPIN1          PPTSVMTRLILIMVWRKLIRNPNTYSSLFGLTWSLVSFRWNLKMPAIIAQSISILSDAGL 507
AtPIN1          PPTSVMTRLILIMVWRKLIRNPNSYSSLFGITWSLISFKWNIEMPALIAKSISILSDAGL 515
StPIN7          PAASVMTRLILIMVWRKLIRNPNTYSSLLGLAWSLISFRWNIQMPLIFAKSISILSDAGL 480
StPIN9          PPASVMTRLILIMVWRKLIRNPNTYSSLIGLTWSLVSFKWNVQMPAIIAKSISILSDAGL 487
StPIN3          PPASVMTRLILIMVWRKLIRNPNTYSSLIGLIWSLISYRWHVHMPKIIEKSISILSDAGL 515
StPIN4          PPASVMTRLILIMVWRKLIRNPNTYSSLIGLIWSLISFRWHVHMPKIIEKSISILSDAGL 547
AtPIN3          PPASVMTRLILIMVWRKLIRNPNTYSSLIGLIWALVAFRWHVAMPKIIQQSISILSDAGL 533
AtPIN7          PPASVMTRLILIMVWRKLIRNPNTYSSLIGLIWALVAFRWDVAMPKIIQQSISILSDAGL 508
AtPIN4          PPTSVMTRLILIMVWRKLIRNPNTYSSLIGLIWALVAYRWHVAMPKILQQSISILSDAGL 505
StPIN2          PPASVMTRLILIMVWRKLIRNPNTYASLIGLIWSLVSFRWNIQMPSIVKGSISILSDAGL 524
AtPIN2          PPASVMTRLILIMVWRKLIRNPNTYSSLFGLAWSLVSFKWNIKMPTIMSGSISILSDAGL 540
StPIN8          ------------------------------------------------------------
AtPIN8          RTRSVGTMKILLKAWRKLIINPNTYATLIGIIWATLHFRLGWNLPEMIDKSIHLLSDGGL 260
StPIN6          PNALVMLRLIIVMVGRKLSRNPNTYSSILGLLWSLISFKWNVGMPSLVKYSIKIISDAGL 421
AtPIN6          PSAIVMMRLILTVVGRKLSRNPNTYSSLLGLVWSLISFKWNIPMPNIVDFSIKIISDAGL 393
StPIN5          NNAELAFWPLMKAVSTKLAKNPNSYACFLGLFWALVASRWHFRMPSIIEGSILIMSKAGS 246
StPIN10         KNTTPSLGSVMTIVWTKLSKNPNFYACFLGIMWSLVADRWHFVLPNIVKECISIMSKAGS 240
AtPIN5          VVGEKSFLEVMSLVWLKLATNPNCYSCILGIAWAFISNRWHLELPGILEGSILIMSKAGT 242

StPIN1          GMAMFSLGLFMALQPRIIACGNSVASFAMAVRFLTGPAVMAAASIAVGLRGTLLHVAIVQ 567
AtPIN1          GMAMFSLGLFMALNPRIIACGNRRAAFAAAMRFVVGPAVMLVASYAVGLRGVLLHVAIIQ 575
StPIN7          GMAMFSLGLFMALQPKMISCGKTIAAFSMAVRFISGPAVMAASFAIGLRGVLLHIAIVQ 540
StPIN9          GMAMFSLGLFMALSPRIIACGKTIALFSMGVRFLTGPAVMAAASIAVGLRGVLLHIAIVQ 547
StPIN3          GMAMFSLGLFMALQPKIIACGNTVATFAMAVRFLTGPAVMAAASIIVGLRGTLLHVAIVQ 575
StPIN4          GMAMFSLGLFMALQPKIIACGNTVATFAMAVRFLTGPAVMAAASIAVGLRGTLLHVAIVQ 607
AtPIN3          GMAMFSLGLFMALQPKLIACGNSVATFAMAVRFLTGPAVMAVAAIAIGLRGDLLRVAIVQ 593
AtPIN7          GMAMFSLG----------------ESSFYSVSFFR------------------------- 527
AtPIN4          GMAMFSLGLFMALQPKIIACGNSVATFAMAVRFITGPAIMAVAGIAIGLHGDLLRIAIVQ 565
StPIN2          GMAMFSLGLFMALQPKIIACGKSVATFSMAVRFLTGPAVIAATSIAIGLRGVLLHVAIVQ 584
AtPIN2          GMAMFSLGLFMALQPKIIACGKSVAGFAMAVRFLTGPAVIAATSIAIGIRGDLLHIAIVQ 600
StPIN8          -------GVFMASQASIIACGTKKAILAMALKFVLGPVLMAISSIAVGLRGQLFRLAIVQ 236
AtPIN8          GMAMFSLGLFMASQSSIIACGTKMAIITMLLKFVLGPALMIASAYCIRLKSTLFKVAILQ 320
StPIN6          GMAMFSLGLFMALQPRIIACGTKMATIGMAIRFIGGPLVMSAASIAVGLKGVRLHTAIVQ 481
AtPIN6          GMAMFSLGLFMALQPKMIPCGAKKATMGMLIRFISGPLFMAGASLLVGLRGSRLHAAIVQ 453
StPIN5          GVAMFSMGLFMALQGKIIACGAALTIYAMILRFVVGPATMALGCVVLGLRGNVLRIAIIQ 306
StPIN10         GIGMFTIGVFVAMQQKVMAGGTGVIVFGLFLRFFIGPATMTIGSFVVGLHGNVLRASILQ 300
AtPIN5          GTAMFNMGIFMALQEKLIVCGTSLTVMGMVLKFIAGPAAMAIGSIVLGLHGDVLRVAIIQ 302
                       *                      : *.

StPIN1          AALPQGIVPFVFAKEYNVHPDILSTGVIFGMLIALPITLVYYIFMGL-- 614
AtPIN1          AALPQGIVPFVFAKEYNVHPDILSTAVIFGMLIALPITLLYYILLGL-- 622
StPIN7          AALPQGIVPFVFAKEYSLHPDILSTGVIFGMLIALPITLVYYILLGL-- 587
StPIN9          AALPQGIVPFVFAKEYGVHPDILSTGVIFGMLVALPITLVYYILLGL-- 594
StPIN3          AALPQGIVPFVFAKEYNVHPAILSTX----------------------- 601
StPIN4          AALPQGIVPFVFAKEYNVHPAILSTAVIFGMLIALPITLVYYIILGL-- 654
AtPIN3          AALPQGIVPFVFAKEYNVHPAILSTGVIFGMLIALPITLVYYILLGL-- 640
AtPIN7          -------------------------------------------------
AtPIN4          AALPQGIVPFVFAKEYNVHPTILSTGVIFGMLIALPITLVYYILLGL-- 612
StPIN2          AALPQGIVPFVFAKEYNLHPDILSTAVIFGMLVALPITILYYVLLGV-- 631
AtPIN2          AALPQGIVPFVFAKEYNVHPDILSTAVIFGMLVALPVTVLYYVLLGL-- 647
StPIN8          AALPQGIVPFVFAKEYNIHPTILSTGVIFGMLIAIPIALAYYFLLEI-- 283
AtPIN8          AALPQGVVPFVFAKEYNLHPEIISTGVIFGMLIALPTTLAYYFLLDL-- 367
StPIN6          AALPQGIVPFVFAREYGLHPDILSTGVIFGMLVSLPVTLLYYVLLGL-- 528
AtPIN6          AALPQGIVPFVFAREYNLHPDLLSTLVIFGMIVSLPVTILYYVLLGL-- 500
StPIN5          AALPQAVTSFVYAQEYGLHADVLSTAVIVGTIISLPLLIAYYAILDIMP 355
StPIN10         VILSPYSLSFLNHTEY--------IVIII-------------------- 321
AtPIN5          AALPQSITSFIFAKEYGLHADVLSTAVIFGMLVSLPVLVAYYAALEFIH 351
```
